# Books and Magazines

**Published:** 1905-12

**Authors:** 


### Books and Magazines.

Anatomy and Physiology for Nurses. By LeRoy Lewis, M. D., Surgeon to and lecturer on Anatomy and Physiology for Nurses at the Lewis Hospital, Bay City, Mich. 12mo of 312 pages, with 100 illustrations. Philadelphia and London: W. B. Saunders & Company, 1905. Cloth, $1.75, net.

Dr. Lewis has based the plan and scope of his excellent little work for nurses on the methods he employs in teaching these subjects. His object was so to deal with anatomy and physiology that the student, while grasping the primary principles, would at the same time, lay a broad foundation for a wider study. The text, therefore, is simple and comprehensive. It would not be just if in giving our unstinted praise to this work we neglected to mention the illustrations. Evidently, Dr. Lewis is thoroughly familiar with the needs of the trained nurse along these lines. Every illustration selected, and a number of them are in colors, has been chosen for a definite purpose, which it admirably fulfills. For a work of its size it contains an immense amount of information, and is just the kind desired, presented in the right way.

---

Practical Massage in Twenty Lessons. By Hartvig Nissen, Instructor and Lecturer in Massage and Gymnastics at Harvard University Summer School; Director of Physical Training, Brookline Public Schools; Former Acting Director of Physical Training, Boston Public Schools; Former Instructor of Physical Training at Johns Hopkins University and Wellesley College; Former Director of the Swedish Health Institute, Washington, D. C., etc., etc. Author of "Swedish Movement and Massage Treatment," "A, B, C of Swedish Educational Gymnastics," "Rational Home Gymnastics," etc. With 46 original illustrations. 168 pages. Price, extra cloth, $1, net. F. A. Davis Company, publishers, 1914-16 Cherry street, Philadelphia.

The value of massage is so generally recognized that no doctor is excusable for not having a practical knowledge of it. This little book is just what he wants.

---

A Treatise on Diseases of the Skin.—For the use of advanced students and practitioners. By Henry W. Stelwagon, M. D., Ph. D., Professor of Dermatology, Jefferson Medical College, Philadelphia; and Clinical Professor of Dermatology, Woman's Medical College, Philadelphia. Fourth edition, revised. Handsome octavo of 1135 pages, with 258 text-illustrations, and 32 full-page lithographic and half-tone plates. Philadelphia and London: W. B. Saunders & Company, 1905. Cloth, $6, net; sheep or half Morocco, $7, net.

Four large editions of Dr. Stelwagon's work have been required in three years. Surely such a sale bespeaks a book of unusual merit. Notwithstanding the frequency of editions, Dr. Stelwagon has not lost opportunity to bring his book up to the latest knowledge. The therapeutic use of the Roentgen rays, high-frequency current, and Finsen light have been accorded the increased attention their growing importance deserves. We notice the addition of new text-cuts, some thirty-eight in number, and six additional insert plates, all up to the high standard set by the text. The author, by the judicious elimination of redundant material, has kept the size of his book much as before, the increase being some twenty pages. Indeed, it is remarkable the epigrammatic way that Dr. Stelwagon has of saying things—a style most desirable both in a textbook and a reference work for the busy practitioner.

---

The Physicians' Visiting List for 1906. Published by P. Blakiston's Son & Co., Philadelphia, is a beauty. For twenty-five patients a week, $1; for fifty patients, $1.25; perpetual edition No. 1, 1300 names, $1.25.

This book is too well known to require further description. Soft black morocco and gilt edges.

---

A Manual of Diseases of Infants and Children. By John Ruhräh, M. D. Clinical Professor of Diseases of Children, College of Physicians and Surgeons, Baltimore. 12mo volume of 404 pages, fully illustrated. Philadelphia and London: W. B. Saunders & Company, 1905. Flexible leather, $2, net.

Dr. Ruhräh is to be congratulated upon the production of a manual that presents the subject of pediatrics in such a clear yet concise manner. He has outlined the therapeutics of infancy and childhood in a way that can not fail to make for this work a place of first importance in its field. He has given explicit instructions for dosage and prescribing, and a number of useful prescriptions are appended. Infant feeding is given in detail. All the illustrations are practical, and include three inserts. A very valuable feature consists in the many references to pediatric literature so selected as to be easily accessible by the student, enabling him to ascertain the sum of knowledge on any given disease. We give Dr. Ruhräh's work our unqualified recommendation.

---

Coakley's Laryngology.—A Manual of Diseases of the Nose and Throat. By Cornelius G. Coakley, A. M., M. D., Professor of Laryngology in the University and Bellevue Hospital Medical College; Laryngologist to Columbus Hospital, etc., New York. New (3d) edition, revised and enlarged. In one 12mo volume of 594 pages, with 118 engravings and 5 colored plates. Cloth, $2.75, net. Lea Brothers & Co., Publishers, Philadelphia and New York, 1905.

The call for three editions in six years shows that this work is firmly planted in the esteem of the profession. It is a widely prescribed text-book, it answers the need of general practitioners who do throat work and it is a valuable reference manual for specialists.

The author has made a very judicious selection of knowledge from the vast range of laryngological lore. He insists on the importance of thorough diagnosis and furnishes sufficient guidance in all its steps up to and including the final settlement of any doubts by means of microscopic and bacteriological tests. A departure of obvious value has been made in dealing with treatment. From among the multiplicity of medicinal and operative measures the author has chosen those which in his experience have proved the best and has given full details for the benefit of those who have not had the advantage of personal and clinical instruction. The revision for this new edition has been very thorough, bringing the book fully to date in all departments. For convenience a special chapter has been added on Therapeutics, wherein will be found a classification of drugs according to their local action, and a number of useful prescriptions, together with indications for their employment. Reference to these additional remedies has been indicated in the index under the *treatment* of each disease. The excellent series of illustrations has been revised and enriched on a parity with the improvements in the text.

---

Physicians' Pocket Account Book. By J. J. Taylor, M. D. Published by The Medical Council, Philadelphia. Price, $1. 216 pages.

A lot of timely "dont's" inserted in the back of the book as a reminder to the doctor. We have received a copy and find it very handy.

---

Dietetics for Nurses. By Julius Friedenwald, M. D., Clinical Professor of Diseases of the Stomach in the College of Physicians and Surgeons, Baltimore; and John Ruhrah, M. D., Clinical Professor of Diseases of Children in the College of Physicians and Surgeons, Baltimore. 12mo volume of 363 pages. Philadelphia and London. W. B. Saunders & Company, 1905. Cloth, $1.50 net.

"Dietetics for Nurses" has been written on the same practical lines as the larger work on Diet by the same authors. It has been prepared both to meet the needs of the training school and to serve as a ready reference book for the nurse when on a case. The essentials of dietetics are given in a concise, clear manner, and the physiology of digestion with the various classes of foods and the part they play in nutrition have been carefully reviewed. The subjects of infant feeding and the feeding of the sick have been fully discussed, and a brief outline has been given of the principles involved in the nourishment of patients suffering from the various

IN ANY CONDITION

of impaired health characterized
by a tendency to general
exhaustion, the patient who takes

GRAY'S Glycerine TONIC Comp.

regularly will eat more,
sleep better, be less nervous,
have more strength, be less dragged out

THE PURDUE FREDERICK CO.,
298 Broadway, New York diseases in which diet plays an important rôle in treatment. A very useful feature consists in the extensive diet lists, with instructions, enabling the nurse to comprehend and intelligently to carry out the orders of the physicians. Another commendable feature is the large number of recipes for the invalid's dietary. Altogether, it is an excellent little work indispensable to the well-trained nurse.

---

A TEXT-BOOK OF PHYSIOLOGY: FOR MEDICAL STUDENTS AND PHYSICIANS. By Wm. H. Howell, Ph. D., M. D., LL. D., Professor of Physiology, Johns Hopkins University, Baltimore. Octavo volume of 905 pages, fully illustrated. Philadelphia and London: W. B. Saunders & Company, 1905. Cloth, $4, net; half Morocco, $5, net.

Dr. Howell's many years of experience as a teacher of physiology in several of the leading medical schools is evident throughout the entire work in the simple and clear style and in the practical handling of his subject. The author has laid main emphasis upon those facts and views which will be directly helpful in the study of general pathology and in the practical branches of medicine. At the same time, however, we are gratified to see that Dr.

Howell has not ignored the experimental side of the subject. This we consider very important, for it has been through individual research that all the great advances in physiological knowledge have been made. The entire literature of physiology has been thoroughly digested and the important views and conclusions incorporated. Indeed, the author has prepared a text-book which, while preserving the scientific spirit, is at the same time simple and modern in presentation. Every notable advance in physics or chemistry as influencing physiology has been carefully noted. Illustrations have been most freely used, greatly helping in understanding and supplementing the descriptions in the text. Especially valuable are those illustrations employed to make clear the more intricate anatomic physiologic mechanisms. Altogether, we consider it a very valuable book, because it is accurate, up-to-date, and highly practical.

---

A Treatise on Diagnostic Methods of Examination. By Prof. Dr. H. Sahli, of Bern. Edited, with additions, by Francis P. Kinnicutt, M. D., Professor of Clinical Medicine, Columbia University, N. Y.; and Nath'l Bowditch Potter, M. D., Visiting Physician to the City Hospital and to the French Hospital; and Consulting Physician to the Manhattan State Hospital, N. Y. Philadelphia and London: W. B. Saunders & Company, 1905. Octavo of 1008 pages, profusely illustrated. Cloth, $6.50, net; half Morocco, $7.50, net.

We have been anxiously awaiting the publication of Dr. Sahli's great work in English. Its immediate success in Germany will certainly be repeated in this country, and the English-speaking profession owe to Messrs. W. B. Saunders & Company a debt of gratitude for their enterprise. Not only does the distinguished professor exhaustively consider all methods of examination for the purpose of diagnosis, but the explanations of clinical phenomena are given and discussed from physiologic as well as pathologic points of view, and with a thoroughness never before attempted in any clinical work. The examinations of the stomach, sputum, feces, urine, and blood are exhaustively treated. There is an article from the pen of Dr. Theodore C. Janeway giving a brief review of the investigations of American and English observers upon the value of the clinical estimation of blood-pressure, with a description of some newly devised instruments. Some of the new features in the chapter on urine examination are: Seliwanow's reaction for levulose, Bial's test for pentoses, and quantitative determination of urochrome after Klemperer. Osmotic pressure and cryoscopy of the urine are also discussed at length, and a description is given of Liebermann and Posner's method of staining urinary pigments. In the chemical examination much attention is directed to describing methods; and this is done so exactly that it is possible for the clinician to work according to these directions. The nervous system has been very elaborately detailed, giving unusual space to electrical examination. Indeed, the American edition of this great work contains all the material of the new fourth German edition, with which it simultaneously appeared. Many new illustrations have been added by the editors. The work is indispensible to the practitioner.

---

WE HAVE just received from W. B. Saunders & Company, of Philadelphia, the widely-known medical publishers, an unusually attractive illustrated catalogue of their complete list of publications. It seems to us, in glancing through this catalogue, that a list of the Saunders' authors is a census of the leading American and foreign authorities in every branch and specialty of medical science. And new books are being added and new editions issued with a rapidity that speaks well for the success and progressiveness of the house. While comparisons are always odious, still we feel it but justice to say that, in the presentation of facts about the books listed that a probable buyer wishes to know, and also for beauty and durability of mechanical get-up, this catalogue surpasses anything we have heretofore seen. It is truly representative of the house. We understand a copy will be sent free upon request.

